# The impact of positive psychology counseling on sexual and marital satisfaction and anxiety among reproductive-aged women during the COVID-19 pandemic: a randomized controlled clinical trial

**DOI:** 10.1186/s40359-024-01826-2

**Published:** 2024-05-29

**Authors:** Mahdie Arab Bafrani, Roghaiyeh Nourizadeh, Sevil Hakimi, Seyed Alireza Mortazavi, Esmat Mehrabi

**Affiliations:** 1 Department of Midwifery, Faculty of Nursing and Midwifery, Tabriz University of Medical Sciences, Tabriz, Iran; 2 Department of Midwifery, Faculty of Health Science, EGE university, Izmir, Turkey; 3 Daman Clinic of Psychology, Tehran, Iran

**Keywords:** Positive counseling, Marital satisfaction, Sexual satisfaction, Anxiety, COVID-19 pandemic

## Abstract

**Background:**

Sexual and marital satisfaction is considered one of the important factors in happiness and life satisfaction of couples. COVID-19 pandemic results in psychological effects, such as increased anxiety levels which can affect sexual and marital satisfaction. This study aimed to investigate the impact of positive psychology on women’s sexual and marital satisfaction.

**Methods:**

A randomized controlled trial was conducted on 72 married women of reproductive age in Tabriz, Iran between February 2021 and May 2022. The participants were randomly divided into the intervention and control groups. There was no significant difference between the control and intervention groups in terms of the socio-demographic characteristics (*p* < 0.05). The mean age of the participants in the intervention and control groups was 31.8 ± 6.92 and 30.97 ± 5.09 years, respectively. The intervention group attended seven 60–90 min counseling sessions at weekly intervals. The Spielberger anxiety, sexual satisfaction and marital satisfaction questionnaires were completed before and four weeks after the intervention.

**Results:**

The results of this study indicated that after counseling, the average overall score of marital satisfaction [MD: 15.46, 95% CI: 7.47 to 23.41, *p* = 0.034] and sexual satisfaction [MD: 7.83, 95% CI: 6.25 to 9.41, *p* = 0.001] significantly increased in the intervention group compared to the control group. Also, the mean score of state anxiety [MD: -2.50, 95% CI: -4.19 to -0.80, *p* = 0.001] and trait anxiety [MD: -1.03, 95% CI: -2.46 to -0.09, *p* = 0.032] significantly decreased after counseling in the intervention group compared to the control group.

**Conclusions:**

Using counseling based on a positive psychology approach can improve anxiety, sexual and marital satisfaction, and anxiety of women of reproductive age during the COVID-19 pandemic. However, further randomized clinical trials are needed before making a definitive conclusion.

**Trial registration:**

Iranian Registry of Clinical Trials (IRCT): IRCT20171007036615N8. Date of registration: 11/28/21. Date of first registration: 11/28/21. URL: https://www.irct.ir/user/trial/58680/view; Date of recruitment start date: 12/01/21.

## Background

Marital satisfaction is one of the common concepts used to evaluate marital happiness and stability. In fact, the success of marriage and couple’s satisfaction with their married life is more important than the marriage itself [1]. Marital satisfaction refers to a condition in which couples meet each other’s needs and the partner is understood, supported, and approved [2]. In general, sexual satisfaction and marital compatibility are positive and enjoyable attitudes arising from various aspects of marital relations [3]. On the other hand, marital relations are the main source of social support for most couples, acting as a protective factor against psychological and physiological injuries [4] An increase in marital satisfaction leads to the enhancement of sexual and marital satisfaction, marriage stability and consolidation of the family foundation, and even improvement of the quality of life, and success in work and social relations [4–7]. Marital satisfaction can lead to the fulfillment of many physical and psychological needs, and in case of dissatisfaction, couples and especially children will be encountered to severe psychological injuries [8]. Also, the decrease in marital satisfaction can cause dissatisfaction with sexual relations and the couple’s decision to divorce [9, 10]. Sexual and marital satisfaction are influenced by factors, such as individual variables, including women’s sexual knowledge and awareness, sexual disorders, personality traits, physical illness, and mental problems, such as anxiety and depression [11] The results of early studies revealed that sexual and interpersonal factors, communication factors, and mental health have a significant effect on marital satisfaction [12].

Global crises, such as Covid-19 are known to increase fear and anxiety, create significant disruption in daily functioning, and threaten mental health [13, 14]. During the last few years, Covid-19 pandemic has been associated with many psychological consequences, of which anxiety is one of the most common [15]. Based on the results of some studies, COVID-19 pandemic and long-term quarantine lead to the occurrence and increase of mental disorders, such as depression, anxiety, stress, post-traumatic stress, anger, and feelings of social isolation [16–20]. Finally, the high level of chronic stress reduces factors, such as libido [21], satisfaction with sexual life [15–22], and marital satisfaction [23], and leads to an increase in sexual dysfunction of couples [24, 25]. Therefore, the fear of COVID-19 contraction, long-term quarantine, and relatively high mortality rate caused anxiety and decreased satisfaction in relations [26, 27].

Based on the findings of psychological research, positive emotions play a significant role in the psychological recovery process of people experienced severe stress or suffered from mental disorders, such as anxiety and depression [28]. Positive psychology is a new domain of psychology that was officially founded by Professor Martin Seligman (2000), the president of the American Psychological Association. Seligman et al. believed that positive psychology is a scientific study of positive experiences and positive personal traits, and the institutions facilitating their development and seeking to improve the quality of life of people and prevent psychological damage caused by a fruitless and meaningless life [29]. The effectiveness of positive psychology-based interventions by focusing on concepts, such as gratitude, personal skills and capabilities, expressing emotions and feelings, effective communication, hope and happiness on sexual satisfaction [30], women’s marital satisfaction [25], and stress, anxiety, and depression [31] are demonstrated in past studies. However, the issues related to sexual health and fertility in times of crisis should be given special attention, since they are closely related to general health and quality of life [22].

The Covid-19 pandemic led to the prevalence of psychological complications and an increase in the level of anxiety in society [19, 32]. In the same vein, sexual and marital satisfaction is influenced by psychological factors [33], as conducted studies indicated the adverse effect of the COVID-19 pandemic on these outcomes [12]. Likewise, marital and sexual dissatisfaction affects the common life by reducing the quality of sexual life and disrupting the relationship of couples [10]. Therefore, psychological factors should be regarded to provide counseling for improving sexual and marital satisfaction. Given the adverse psychological effects of the Covid-19 pandemic in recent years and the lack of studies in this field, the present study aimed to evaluate the effect of positive psychology-based counseling on sexual and marital satisfaction and women’s anxiety during the Covid-19 pandemic.

## Methods

### Study design and participants

A randomized controlled trials (RCTs) are considered the highest level of evidence to establish causal associations in clinical research [34]. This type of study is a definitive tool for the evaluation of the effectiveness of an intervention and can establish a cause-and-effect relationship between an intervention and an improved disease outcome [35]. This RCT was conducted on 72 women referring to health centers in Tabriz, Iran from February 2021 to May 2022. The inclusion criteria were married women at reproductive age of 18–49 years old, having a surviving spouse, being sexually active, anxiety score from 43 to 53, sexual satisfaction score less than 100, and not receiving individual counseling services during the participation in the therapy sessions. The exclusion criteria included having a history of physical diseases influencing sexual desire (such as having organ damage, suffering from a chronic physical disease, etc.), having physically debilitating diseases (such as cancer, MS, etc.), stressful variables (such as disability or illness of a family member, such as cancer, retardation, etc.), the wife, the spouse or one of the family members mental illnesses based on the patient’s health record, the occurrence of recent unfortunate events and acute stressful problems (such as the death of a first-degree family member in the last few months, such as death of a child, father, etc.), smoking, the use of alcohol and drugs and other drugs influencing an individuals’ body and mind, taking drugs reducing libido (antidepressants: fluoxetine, sertraline, and paroxetine, antihistamines: chlorpheniramine, drugs affecting blood pressure: clonidine, captopril, benzodiazepines, such as alprazolam (Xanax), etc.), premature menopause and marital discord affecting sexual relations (such as severe marital discords leading to the petition for divorce).

### Sample size

The sample size was calculated 36 per group based on the mean score of marital satisfaction in the study of Vojdani et al. (2014) [25] and considering m_1_ = 32.36, m_2_ = 42.18, sd_1_ = 14.22, and sd_2_ = 13.37, and two-sided α = 0.05, power = 80%, with the assumption of 20% attrition. Based on the variable of sexual satisfaction in the study of Gheshlaghi et al. (2012) [36] and considering m_1_ = 55.48, m_2_ = 61.03, with the assumption of 20% increase in the mean score of sexual satisfaction, sd_1_ = 14.13, sd_2_ = 13.47, and two-sided α = 0.05, and power = 80%, the sample size was estimated to be 15 per group. Owing the anxiety variable in the study of Chan et al. (2004) [37], regarding m_1_ = 45.56, with the assumption of 20% attrition in the anxiety score after the intervention, m_2_ = 36.45, SD_1_ = SD_2_ = 11.16, power = 90%, and Two-sided test, with the assumption of 20% attrition, the final sample size was obtained 34 in each group.

### Sampling and random assignment

This clinical trial was conducted on 72 women of reproductive age of 18 to 49 years old referring to health centers in Tabriz, Iran. Sampling was done after obtaining permission from the ethics committee of Tabriz University of Medical Sciences (IR.TBZMED.REC.1400.677) and registration of the study in the Iranian Registry of Clinical Trials (IRCT code: IRCT20171007036615N8, Date of first registration: 11/28/21).

The city of Tabriz has 92 health centers and the details of all women of reproductive age, including phone numbers and addresses, are available in the web-based system (SIB System). The researcher attended the health centers and extracted the list of all married women of reproductive age along with their phone numbers and addresses. Then, all women were called and briefly explained the objectives and method of the study, and those who were willing to participate in the study were requested to be present at the health center at a certain time. In the first introductory session, the research objectives and method were fully explained, the inclusion criteria were examined, and eligible women signed the written informed consent form to participate in the research. Then, the Spielberger anxiety questionnaire and Larson’s sexual satisfaction questionnaire were completed and women with anxiety scores of 43–53 and sexual satisfaction scores less than 100 were included in the study. ’Other pretest questionnaires, including socio-demographic and obstetric characteristic questionnaire and Enrich’s marital satisfaction questionnaire were also completed by the participants before the intervention (in the first session( by interview. The participants were assigned into the intervention and control groups with a ratio of 1:1 by block randomization using random allocation software (RAS) with a block size of 4 and 6. Block randomization was done by a non-involved person in the sampling and data analysis. The type of allocation was written on a piece of paper and put in opaque envelopes numbered in consecutive order for allocation concealment. A non-involved person in the sampling process opened the envelopes in the order in which the participants entered the study.

### Intervention

Seven counseling sessions (three face-to-face and four virtual sessions) in a group of 3 women for 90 min and in compliance with health protocols to prevent COVID-19 infection, such as masks, disinfectants, and disposable gloves, were held once a week for seven consecutive weeks by the researcher (MSc student in midwifery counseling and certified positive counseling workshop) for the intervention group in the health education hall of the nearest health center to the participants’ residence. Furthermore, the control group attended four counseling sessions (two face-to-face sessions and two virtual sessions) regarding self-care methods for breast and cervical diseases for 60–90 min. In addition, women with an anxiety score in the severe anxiety category were referred to a psychiatrist.

The summary of the content of counseling sessions is presented in the following table (Table 1):

**Table 1:** The contents of the sessions

Educational sessions	Title of the session	Educational content
First	Orientation	Introducing and initial familiarizing, explaining study objectives, titles, work process and framework of positive psychotherapy, role of therapist and responsibilities of clients, introducing three paths leading to happiness and relief of depression (pleasure, commitment, and meaning)
Second	Commitment	**Determining specific capabilities**: Reviewing the specific capabilities of people, avoiding naming and labeling, emphasizing positive activities with immersion **Assignment**: Expressing at least three special capabilities and the help these capabilities have given to people in the past
Third	Commitment/Pleasure	**Developing special capabilities and positive emotions**: The development of specific capabilities, preparation of clients to form specific, objective, and attainable behaviors to develop specific capabilities, training to use the skills of positive adaptation strategies (problem-solving) and help to strengthen and express positive feelings and beliefs to reduce anxiety and worry, the role of positive emotion in well-being (positive life)
Fourth	Pleasure/ Commitment	**Gratitude**: Explaining the positive consequences of gratitude as lasting gratitude and its effects in common life, stating different ways of expressing gratitude, highlighting good and bad memories with an emphasis on gratitude **Assignment**: writing a letter of appreciation to one of the important and influential people in life
Fifth	Pleasure/ Commitment	**Forgiveness**: Expressing the issues of strengthening self-confidence, self-esteem, and dealing with worries, introducing forgiveness as a powerful tool to move from negative feelings and emotions to neutral and even positive emotions **Assignment**: Writing a letter of forgiveness to the person you feel angry with
Sixth	Pleasure	**Optimism and hope**: explaining the concept of hope in married life, expressing how to overcome obstacles to achieving the goals of married life, thinking about important failures in life, emphasizing hope, optimism and gaining positive knowledge **Assignment**: Expressing alternative solutions when you face a problem
Seventh	Commitment/meaning	Training to improve positive social relationships and happiness in life, Active Constructive Responding (ACR) technique, relationship building skills and factors affecting it, proper listening training, listening techniques, reflection, paying attention to body language, summarizing and answering members, questions, conducting the post-test and thanking the participants

### Data collection tools

The data were collected using the questionnaire of socio-demographic characteristics, ENRICH Marital Satisfaction (EMS) Scale, Larson’s sexual satisfaction questionnaire, and Spielberger State-Trait Anxiety Inventory (STAI) before and 4 weeks after intervention by both control and intervention groups. The socio-demographic questionnaire included questions about age, age at marriage, occupation, level of education, history of illness and drug use in the individual and spouse, number of children, income sufficiency, duration of marriage, history of infertility and contraception method.

### Larson’s sexual satisfaction questionnaire

Larson’s sexual satisfaction questionnaire [38], developed by Larson et al. (1998), was used to check sexual satisfaction, as the higher the score, the more sexual satisfaction. This instrument composed of the components of willingness to have sex, sexual attitude, and sexual compatibility with 25 items, scored on a 5-point Likert scale, ranging from 1 to 5 (never = 1, rarely = 2, sometimes = 3, most of the time = 4, and always = 5). The scores range from 25 to 125 and sexual satisfaction is classified into the levels of no sexual satisfaction (score less than 50), low satisfaction (score between 51 and 75), moderate satisfaction (score between 76 and 100) and high satisfaction (score above 101). The Cronbach’s alpha coefficient of the tool was estimated to be 0.93 in the study of Bahrami et al. [39].

### Enrich marital satisfaction questionnaire

Enrich marital satisfaction questionnaire [40] developed by Olson and Fowers was employed to assess marital satisfaction. This tool includes the dimensions of idealistic distortion, marital satisfaction, communication, and conflict resolution. The items are scored based on a 5-point Likert scale, ranging from 1 to 5 (I completely agree = 5, I agree = 4, neither agree nor disagree = 3, I disagree = 2, and I completely disagree = 1). The total score range is between 35 and 175. A score of 0–15%,16–35%, 36–60%, 61–80%, and 81–100% represents high dissatisfaction, somewhat dissatisfaction, somewhat happiness, high happiness, and high satisfaction with all aspects of marital relations, respectively. Soleimanian [41] investigated the psychometrics of this questionnaire in Iran and its reliability was reported to be 0.9 using Cronbach’s alpha coefficient.

#### Spielberger state-trait anxiety inventory (STAI)

The STAI includes 40 items in two parts. The first part measures state anxiety and includes 20 items scored on a 4-point Likert scale (never = 1 to always = 4). The second part of STAI assesses trait anxiety, consisting of 20 items (21–40) scored based on a 4-point Likert scale (almost never = 1 to almost always = 4). Scores of state and trait anxieties are calculated separately and summed [42]. The total score range is between 20 and 80 for items 1 to 20 of state and trait forms. This questionnaire has been validated in Iran and its Cronbach’s alpha has been reported as 0.91 and 0.90 for state and trait anxiety, respectively [43].

### Data analysis

Data were analyzed using SPSS version 26 software. The normality of quantitative data was checked using the Kolmogorov-Smirnov test and all data had a normal distribution. The socio-demographic characteristics were compared through independent t-test and Chi-square test. The independent t-test was used before the intervention and ANCOVA by adjusting the baseline score was applied four weeks after the intervention to compare the mean scores of marital and sexual satisfaction and state and trait anxiety and *p* < 0.05 was considered significant.

## Results

**Fig. 1:**
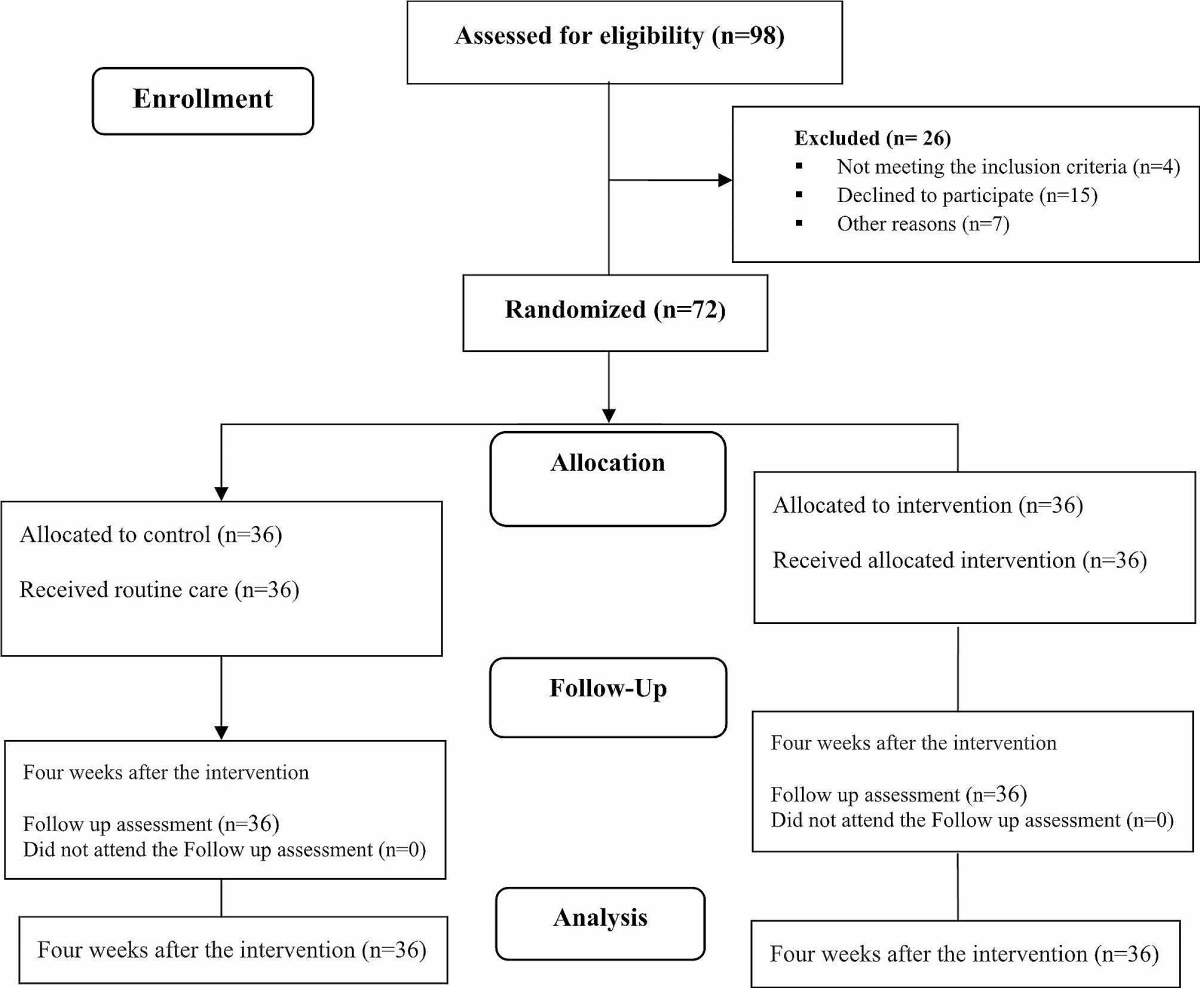
Flowchart of the study

Figure 1 displays the flowchart of the study. After checking the inclusion criteria and willingness to participate in the study, out of a total of 98 women aged 18–45 years, 72 eligible women entered the study and were randomly assigned into the intervention (*n* = 36) and control (*n* = 36) groups. The number of participants remained constant throughout the study and during four four-week follow-up after the sessions ended. There was no significant difference between the control and intervention groups in terms of the socio-demographic characteristics (*p* < 0.05). The mean age of the participants in the intervention and control groups was 31.8 ± 6.92 and 30.97 ± 5.09years, respectively (the participant’s age range was between 19 and 47). The educational level of more than half of the women in the study groups was Bachelor’s and Master’s degree. Furthermore, less than 10% of women from each group did not have a university education. Most of the women in the intervention (61.1%) and control (80.6%) groups were housekeepers. The income level of more than half of the families was almost sufficient and about one-third of the families had completely sufficient income and only 11.1% of women in the intervention group did not have enough income (Table 2).

Before the intervention, there was no significant difference between the two groups in terms of sexual satisfaction (*p* = 0.97). Based on the ANCOVA test by adjusting the baseline score, the mean score of sexual satisfaction in the intervention group was significantly higher than that in the control group, four weeks after the intervention [MD: 7.83, 95% CI: 6.25 to 9.41, *p* = 0.001] (Table 3).

The results revealed that the intervention was effective in increasing the total score of marital satisfaction and all its components, including marital satisfaction, communication, conflict resolution, and ideal distortion. Before the intervention, the results of the independent t-test indicated no significant difference between the total score of marital satisfaction and its components between the intervention and control groups (*p* = 0.63). Four weeks after the intervention, the analysis of the results indicated a significant increase in the total score of marital satisfaction [MD: 15.46, 95% CI: 7.47 to 23.41, *p* = 0.03] (Table 4). Before the intervention, there was no significant difference in the state (*p* = 0.33) and trait (*p* = 0.73) anxiety scores between the intervention and control groups. After the intervention, a significant decrease was observed in the level of state anxiety [MD: -2.50, 95% CI, -4.19 to -0.80, *p* = 0.001] and trait anxiety [MD: -1.03, 95% CI: 2.46 to -0.09, *p* = 0.03] in the intervention group compared to the control group (Table 5).

**Table 2:** The socio-demographic characteristics of participants in the intervention and control groups

Variable		Intervention (*n* = 36) Number (percent)	Control (*n* = 36) Number (percent)	*p*-value
Age (years)		31.08 (6.92)	30.97 (5.09)	0.07
Spouse’s age (years)		35.33 (7.34)	33.05 (5.72)	0.14
Age of marriage (years)	< 20	9 (25.0)	11 (30.6)	0.86
	20–25	22 (61.1)	22 (61.1)	
	26–30	3 (8.3)	2 (5.6)	
	31–35	2 (5.6)	1 (2.8)	
Spouse’s marriage age (years)	20–25	21 (58.3)	16 (44.4)	0.48
	26–30	11 (30.6)	14 (38.9)	
	31–35	4 (11.1)	6 (16.7)	
Education	< Diploma	2 (5.6)	3 (8.3)	0.69
	Diploma/Post Graduate Diploma	12 (33.3)	9 (25.0)	
	Bachelor’s/Master’s	22 (61.1)	24 (66.7)	
Spouse’s education	< Diploma	4 (11.1)	1 (2.8)	0.32
	Diploma/Post Graduate Diploma	8 (22.2)	11 (30.6)	
	Bachelor’s/Master’s	24 (66.7)	24 (66.7)	
Job	Employed	4 (11.1)	1 (2.8)	0.19
	Shopkeeper	6 (16.7)	5 (13.9)	
	Has a night shift	4 (11.1)	1 (2.8)	
	Housewife	22 (61.1)	29 (80.6)	
Spouse’s job	Jobless	1 (2.8)	0 (0.0)	0.79
	Employed	15 (41.7)	16 (44.4)	
	Retired	1 (2.8)	1 (2.8)	
	Other	19 (52.8)	19 (52.8)	
disease background	Yes	4 (11.1)	5 (13.9)	0.72
	No	32 (88.9)	31 (86.1)	
medication	Yes	5 (13.9)	5 (13.9)	1.00
	No	31 (86.1)	31 (86.1)	
Spouse’s disease background	Yes	2 (5.6)	3 (8.3)	0.64
	No	34 (49.4)	33 (91.7)	
Spouse’s medication	Yes	2 (5.6)	2 (5.6)	1.00
	No	34 (49.4)	34 (49.4)	
Drug use by spouse	Cigarette	4 (11.1)	7 (19.4)	0.61
	Hookah	3 (8.3)	3 (8.3)	
	None	29 (80.6)	26 (72.2)	
Income sufficiency	Completely sufficient	10 (27.8)	9 (25.0)	0.10
	Almost sufficient	22 (61.1)	27 (75.0)	
	Never sufficient	4 (11.1)	0 (0.0)	
duration of marriage (year)	< 1	1 (2.8)	4 (11.1)	0.02
	1–5	16 (44.4)	18 (50.0)	
	6–10	4 (11.1)	10 (27.8)	
	11–15	10 (27.8)	2 (5.6)	
	16–20	5 (13.9)	2 (5.6)	
Number of children	0	15 (41.7)	16 (44.4)	0.57
	1	11 (30.6)	14 (38.9)	
	2	9 (25.0)	6 (16.7)	
	> 2	1 (2.8)	0 (0.001)	
Infertility	Yes	2 (5.6)	0 (0.001)	0.15
	No	34 (94.4)	36 (100.0)	
Contraception method	Natural method	22 (61.1)	17 (47.2)	0.14
	Condom	8 (22.2)	17 (47.2)	
	Tablets	3 (8.3)	1 (2.8)	
	IUD	1 (2.8)	1 (2.8)	
	Vasectomy	2 (5.6)	0 (0.001)	

**Table 3:** The comparison of the mean score of sexual satisfaction before and 4 weeks after intervention in the studied groups

Variable	Intervention (*n* = 36) Mean (SD)	Control (*n* = 36) Mean (SD)	AMD (95%CI)	*p*-value
		Sexual Satisfaction		
Before intervention	91.72 (9.74)	94.67 (3.87)	2.94 (-0.54 to 6.43)	0.97^*^
After intervention	100.33 (10.82)	95.44 (3.74)	7.83 (6.25 to 9.41)	0.001^**^

* t-test, ** ANCOVA test with base line control, # adjusted mean difference with 95% confidence interval

**Table 4:** The comparison of the mean score of Marital satisfaction and its components before and 4 weeks after intervention in the studied groups

Variable	Intervention (*n* = 36) Mean (SD)	Control (*n* = 36) Mean (SD)	AMD (95%CI)	*p*-value
	**Marital satisfaction (score range 10–50)**			
Before intervention	35.30 (6.82)	36.86 (6.27)	1.55 (0.47 to 6.63)	0.08^*^
After intervention	39.38 (5.78)	36.19 (5.80)	2.15 (1.19 to 3.11)	0.001^**^
	**Communication (score range 10–50)**			
Before intervention	34.91 (7.10)	35.41 (5.82)	0.50 (0.14 to 1.05)	0.07^*^
After intervention	35.66 (6.65)	34.23 (5.50)	1.92 (1.22 to 2.61)	0.001^**^
	**Conflict resolution (score range 10–50)**			
Before intervention	30.77 (6.52)	34.75 (5.66)	3.97 (1.10 to 6.84)	0.001^*^
After intervention	34.66 (6.12)	32.36 (5.53)	1.40 (0.77 to 2.03)	0.001^**^
	**Ideal distortion (score range 5–25)**			
Before intervention	20.08 (3.06)	20.88 (2.67)	0.80 (-0.54 to 2.15)	0.23^*^
After intervention	20.45 (2.68)	23.98 (2.28)	3.32 (2.36 to 0.71)	0.03^**^
	**Overall marital satisfaction score (score range 35–175)**			
Before intervention	126.08 (20.84)	128.33 (15.77)	2.25 (0.56 to 4.33)	0.63^*^
After intervention	128.91 (18.90)	113.72 (15.39)	15.46 (7.47 to 23.41)	0.03^**^

* t-test, ** ANCOVA test with base line control, # adjusted mean difference with 95% confidence interval

**Table 5:** The comparison of mean score of state and trait anxiety before and 4 weeks after intervention in the studied groups

Variable	Intervention (*n* = 36) Mean (SD)	Control (*n* = 36) Mean (SD)	AMD (95%CI)	*p*-value
		**State Anxiety**		
Before intervention	46.16 (3.00)	45.52 (2.60)	-0.63 (-1.96 to 0.68)	0.33^*^
After intervention	43.55 (5.50)	45.50 (2.63)	-2.50 (-4.19 to -0.80)	0.001^**^
		**Trait Anxiety**		
Before intervention	46.50 (3.76)	46.25 (2.37)	-0.25 (-1.73 to 1.23)	0.73^*^
After intervention	44.02 (3.78)	45.86 (2.39)	-1.03 (2.46 to -0.09)	0.03^**^

* t-test, ** ANCOVA test with base line control, # adjusted mean difference with 95% confidence interval

## Discussion

Positive psychology interventions can be effective in the enhancement of subjective and psychological well-being, as well as in helping to reduce depressive symptoms [44]. The application of topics such as meaning, coping, self-compassion, courage, gratitude, character strengths, positive emotions, positive interpersonal processes, and high-quality communication in positive psychology has also been demonstrated in supporting individuals during a pandemic [45]. Based on the findings, holding counseling sessions with a positive approach can increase the sexual and marital satisfaction of women of reproductive age and reduce their anxiety during the COVID-19 pandemic.

The results showed positive counseling increases women’s sexual satisfaction from medium to high level in the intervention group after receiving counseling. In line with the results of the present study, Salehi Moghaddam et al. [46] examined the effectiveness of the sexual skills training program on women’s sexual satisfaction and the sexual satisfaction score before and after counseling in the intervention group was higher than that in the current study and was in the range of high sexual satisfaction. However, positive counseling resulted in a greater increase in the sexual satisfaction score in the present study. Further, in the study of the effect of mindfulness counseling on improving the sexual satisfaction of women of reproductive age [47], the sexual satisfaction score of 20 women in the intervention group significantly increased after receiving the counseling. The increase in the sexual satisfaction score in the aforementioned study was more than in the present study and the effect of combining the online sessions with face-to-face sessions and the special conditions of the Covid-19 pandemic in the current study are considered as influential factors. Alimohammadi et al. [48] investigated the effectiveness of group counseling based on Bandura’s self-efficacy theory on the sexual satisfaction of newlywed women and reported no statistically significant difference between the two groups in terms of sexual satisfaction (*p* = 0.058), which was inconsistent with the findings of the present study. The researchers revealed that the duration of the follow-up and the limited number of counseling sessions affect the ineffectiveness of the intervention on women’s sexual satisfaction. This study demonstrated the necessity of conducting psychological interventions with diverse and innovative approaches to find the most effective intervention based on the desired outcome. To the best of our knowledge, no study similar to the present study was found during the COVID-19 pandemic in the literature review.

The results of the present study illustrated that positive psychology (optimistic) based counseling was effective in increasing the total score of marital satisfaction and all its components. The highest enhancement was seen in marital satisfaction and conflict resolution, respectively. Actually, positive psychology-based counseling is effective in reducing differences and increasing the ability to control conflicts between couples through creating positive feelings and increasing the capability and self-confidence of the studied women. Finally, the formation of better marital relations and fewer conflicts lead to the improvement of marital satisfaction of couples. In another study, Zahedi et al. [49] compared the effect of acceptance and commitment therapy (ACT) and cognitive-behavioral therapy (CBT) on marital satisfaction of 45 women assigned into the ACT, CBT, and control groups. They indicated that the marital satisfaction of women increased in both intervention groups and ACT-based counseling had a greater impact on women’s marital satisfaction compared to the CBT-based counseling. In a study, Zarei Abolkheir et al. [50] reported that although CBT-based counseling is effective in increasing women’s marital satisfaction, this increase was not statistically significant. The counseling content was provided to the participants in the form of five online audio files for 5 weeks. Uncertainty of the follow-up sessions by the participants can be a reason for the results of the study.

The results of the present study illustrated that positive psychology-based counseling is effective on the state-trait anxiety of women and reduces their anxiety. It is worth mentioning that despite the significant decrease in the anxiety score of women in the intervention group after receiving the intervention, the mean score of state-trait anxiety was still at an average level. In line with the findings of the present study, the investigation of the effect of online psychological interventions, including CBT, stress reduction techniques, mindfulness, and positive counseling on the anxiety of patients with COVID-19 [51] indicated the positive effect of the intervention in reducing the participants’ anxiety. In the same vein, Jinzhi Li et al. [52] denoted the effectiveness of CBT-based counseling on mental health and reducing anxiety in patients with COVID-19. Further, Dincer and Inangil [53] evaluated the effect of Emotional Freedom Techniques (EFT) on the anxiety level of nurses during the COVID-19 epidemic using the Spielberger questionnaire and reported that the intervention led to a decrease in the anxiety score in the intervention group. The state anxiety score in the aforementioned study was reduced more than that in the present study, and trait anxiety was not investigated. The presence of participants from among educated people and medical staff can influence the better results of the study.

In addition, the results of some descriptive studies demonstrated the negative effects of the COVID-19 pandemic on the outcomes investigated in the present study. A study in Spain indicated that quarantine affected the sexual life of half of the people studied (47.7%), especially women [54]. Although the number of sexual relations increased among the participants compared to before, the scores of the sexual performance index of women were higher before the Covid-19 pandemic and the quality of sexual life of people decreased during the Covid-19 epidemic [21]. Researchers in a review study reported that the marital satisfaction of couples decreased during the Covid-19 pandemic and consequently, the couple’s common life is at risk and psychoeducational counseling is required for couples to improve marital adjustment and communication, emotion regulation, conflict management, and problem resolution during the Covid-19 pandemic [55]. The stress inflicted on couples during the COVID-19 pandemic decreases their marital satisfaction level. Therefore, researchers suggested that psychological interventions should be done considering the damage inflicted on the couples during the COVID-19 pandemic [56], indicating the importance and necessity of appropriate educational and counseling interventions to improve couples’ relationships and their mental health level during the COVID-19 pandemic.

Given that mental health and the level of stress and anxiety of people are closely associated with the quality of their marital health [2, 20, 57], women with low sexual and marital satisfaction and high-stress levels were selected as the target population in the present study. Therefore, it is recommended to consider mental health and the quality of marital relations when providing counseling programs during the epidemic of physical diseases. Additionally, based on the results of the conducted studies, the level of knowledge of marital relations and sexual awareness plays an important role in the level of sexual satisfaction [58] and the quality and stability of marital relations of couples [11]. Therefore, it seems that increasing women’s sexual awareness and empowering them in communication skills can be effective in preventing marital disputes and couples’ dissatisfaction.

### Strengths and limitations

One of the strengths of this study is observing all principles of randomized controlled trial, including random allocation and allocation concealment. Also, providing practical exercises in each session for a better understanding of the topics raised is one of the strengths of the study. Standard and valid questionnaires were used in the present study, which their psychometric properties have been assessed in Iran already. The limitation of this study was that it coincided with the coronavirus pandemic and severe restrictions due to social distancing and quarantine for counseling sessions. In addition, it was not possible to blind the participants and data assessor due to the nature of the intervention. Further, other limitations are the relatively small sample size and the short follow-up period. Therefore, it is recommended to conduct randomized clinical trials with larger sample size and longer follow-up period.

## Conclusion

In general, the results indicated that counseling leads to an increase in sexual and marital satisfaction among women. Therefore, counseling techniques, such as positive psychology can be used to improve women’s satisfaction with their marital relations and sex lives, and help women manage their marital problems and disputes and reduce their anxiety in critical and special situations. In addition, family health policymakers should pay more attention to the negative effects of health crisis conditions on the satisfaction of couples and women’s mental health, and provide solutions to improve marital relations and prevent wide-ranging disputes in the family through holding counseling sessions. It is also suggested to create programs to increase the awareness of health care providers about the important role of marital satisfaction in the health of the family and society, and lead health care providers to identify and improve sexual problems. This can be implemented by changing the continuing education programs or adding psychology units to the educational curriculum of universities and using senior experts in counseling in midwifery in health centers. Holding monthly meetings, providing educational sites and group meetings for discussion and counseling with the presence of a positive psychotherapist in person or online are also suggestions that can be implemented in health care centers.

## Data Availability

Data sets used and/or analyzed during the current study are available from the corresponding author upon reasonable request.

## Abbreviations

AMD: Adjusted Mean Difference
MD: Mean Difference
STAI: State-Trait Anxiety Inventory
